# Supplementary figures and images for: An agent-based model of molecular aggregation at the cell membrane
Source: PLoS One. 2020 Feb 7;15(2):e0226825. doi: 10.1371/journal.pone.0226825 (PMC7006917; doi:10.1371/journal.pone.0226825)

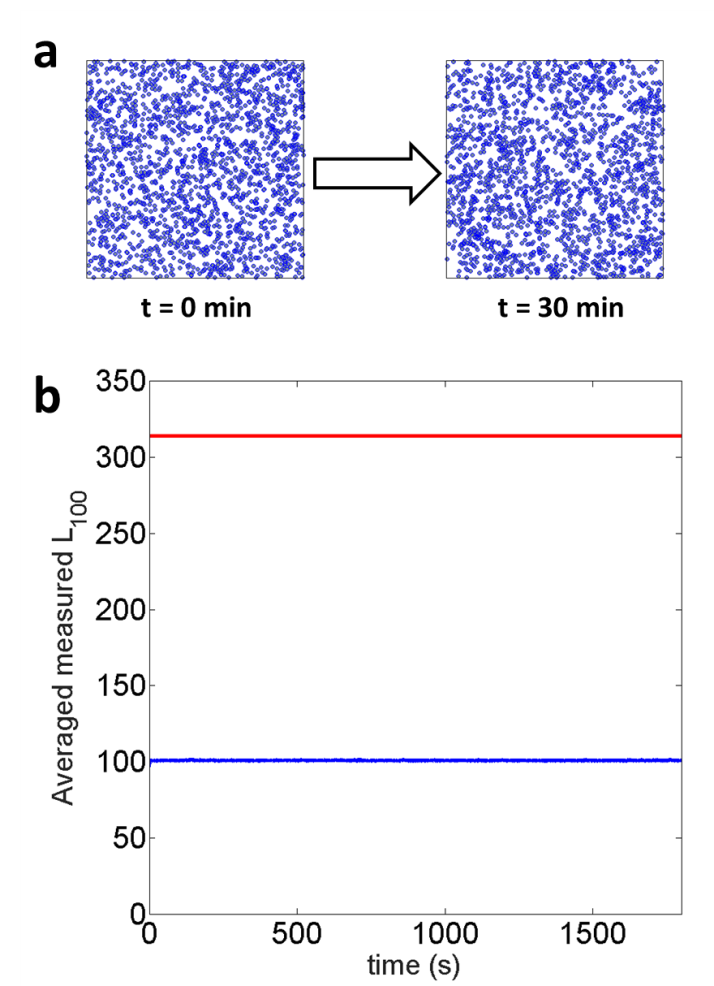

Supplement: S1 Fig — a Molecular distribution from t = 0 min to t = 30 min. b. Resulting <L^100> curve. (TIF) [file pone.0226825.s001.tif]

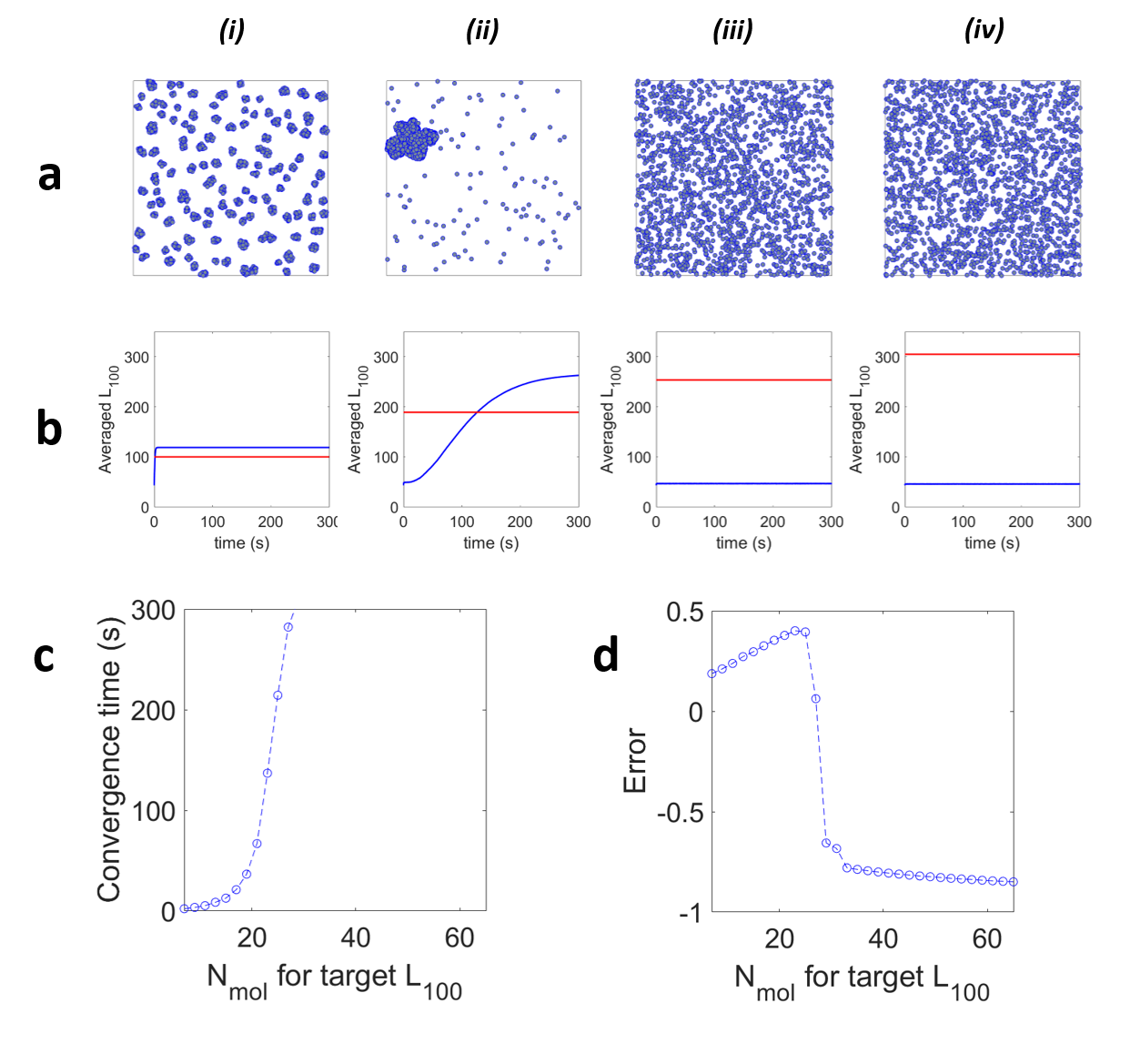

Supplement: S2 Fig — a. Examples of the molecular distributions obtained after 5 min simulations with the target values set at (i) 7, (ii) 25, (iii) 45, (iv) 55, (v) 65 encircled molecules. b. Corresponding <L^100> values extracted from all molecules over time (depicted blue) and their associated fixed targets (depicted red). c. Convergence time and d. Error as a function of the number of molecules for each L100 target (depicted in blue are the values obtained for each target). (TIF) [file pone.0226825.s002.tif]

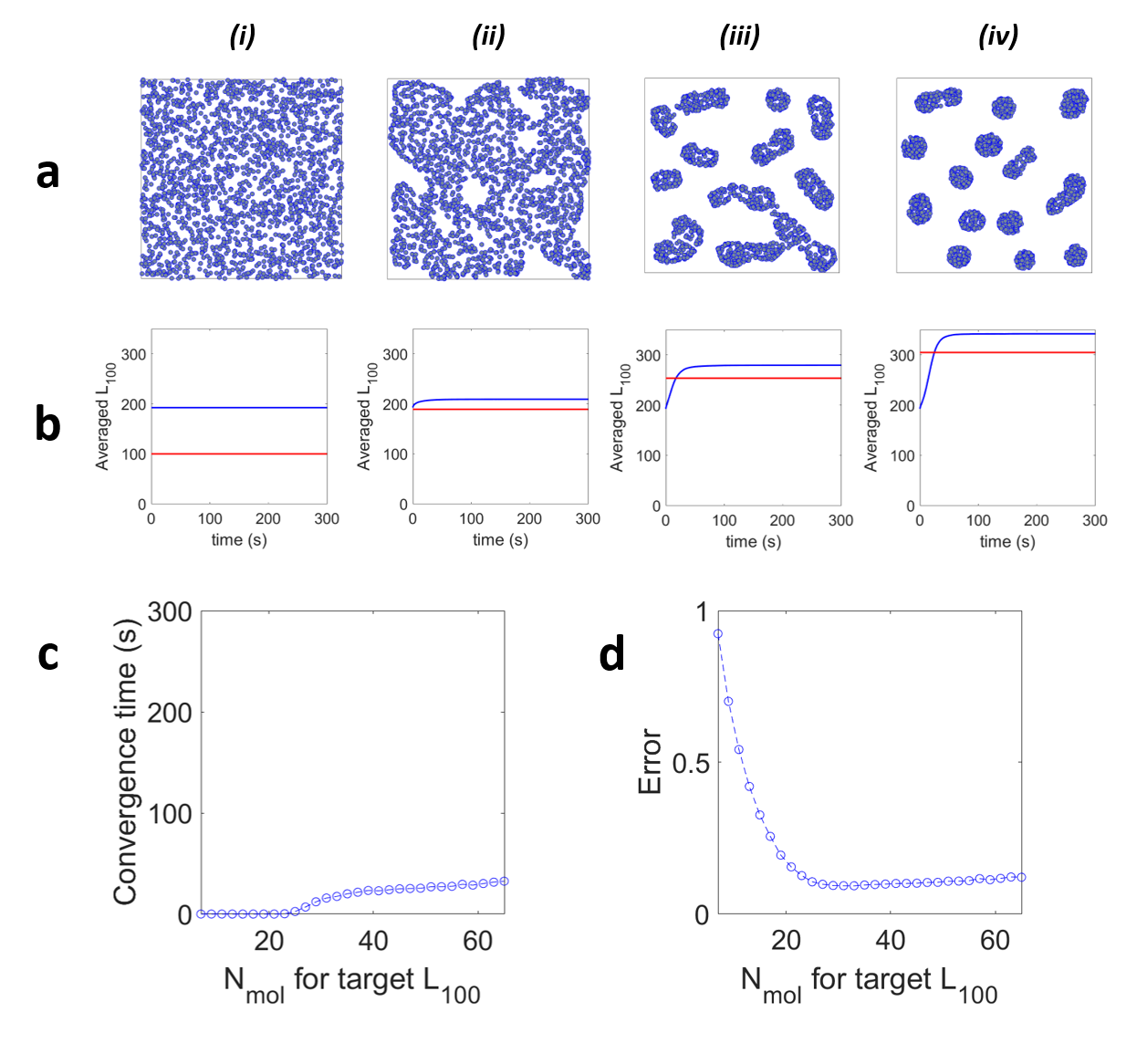

Supplement: S3 Fig — a. Examples of the molecular distributions obtained after 5 min simulations with the target values set at (i) 7, (ii) 25, (iii) 45, (iv) 55, (v) 65, (vi) 80, (vii) 90 encircled molecules. b. Corresponding <L^100> values extracted from all molecules over time (depicted blue) and their associated fixed targets (depicted red). c. Convergence time and d. Error as a function of the number of molecules for each L100 target (depicted in blue are the values obtained for each target). (TIF) [file pone.0226825.s003.tif]

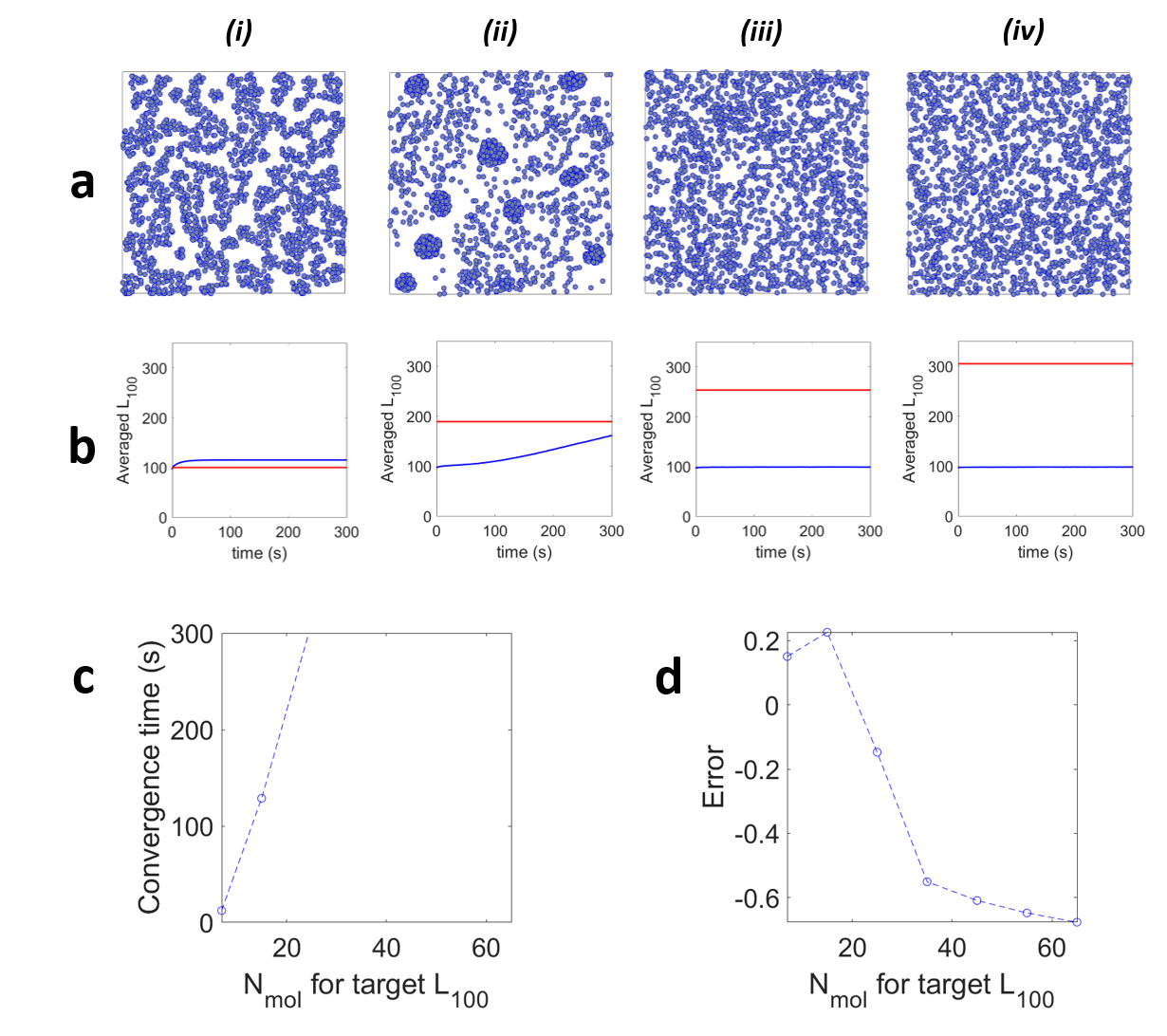

Supplement: S4 Fig — a. Examples of the molecular distributions obtained after 5 min simulations with the target values set at (i) 7, (ii) 25, (iii) 45, (iv) 55, (v) 65 encircled molecules. b. Corresponding <L^100> values extracted from all molecules over time (depicted blue) and their associated fixed targets (depicted red). c. Convergence time and d. Error as a function of the number of molecules for each L100 target (depicted in blue are the values obtained for each target). (TIF) [file pone.0226825.s004.tif]

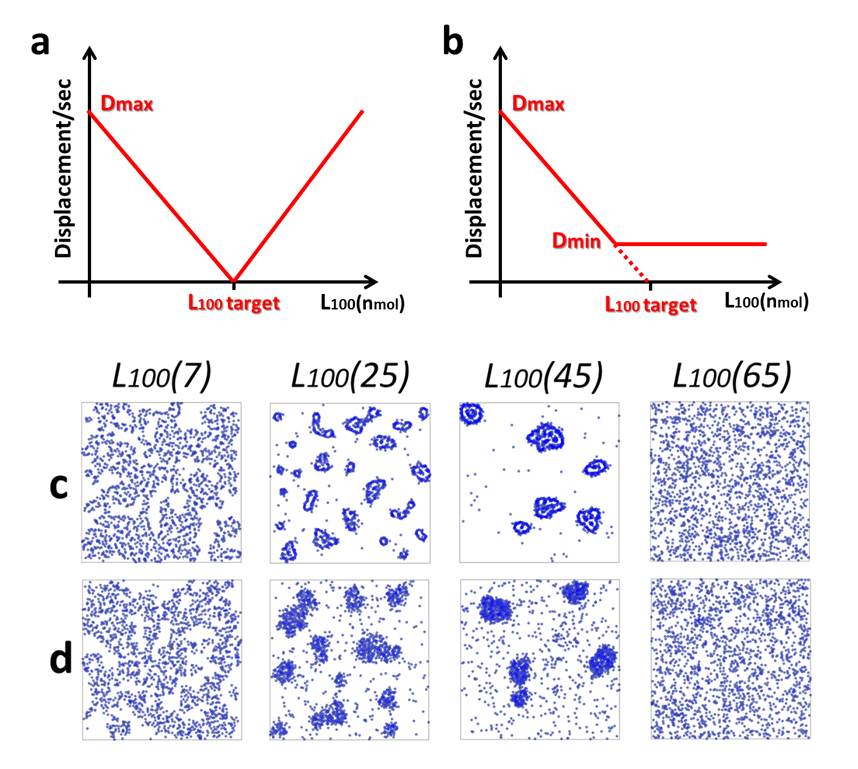

Supplement: S5 Fig — a. Non-converging scenario where a molecule i is immobile for L100,i = L100,target only. b. Converging scenario with Dmin>0. c. Example of final updates frame for target set at 7, 25, 45, 65 encircled molecules, in the non-converging scenario. d. Example of final updates frame for target set at 7, 25, 45, 65 encircled molecules in the converging scenario with Dmin>0. (TIF) [file pone.0226825.s005.tif]

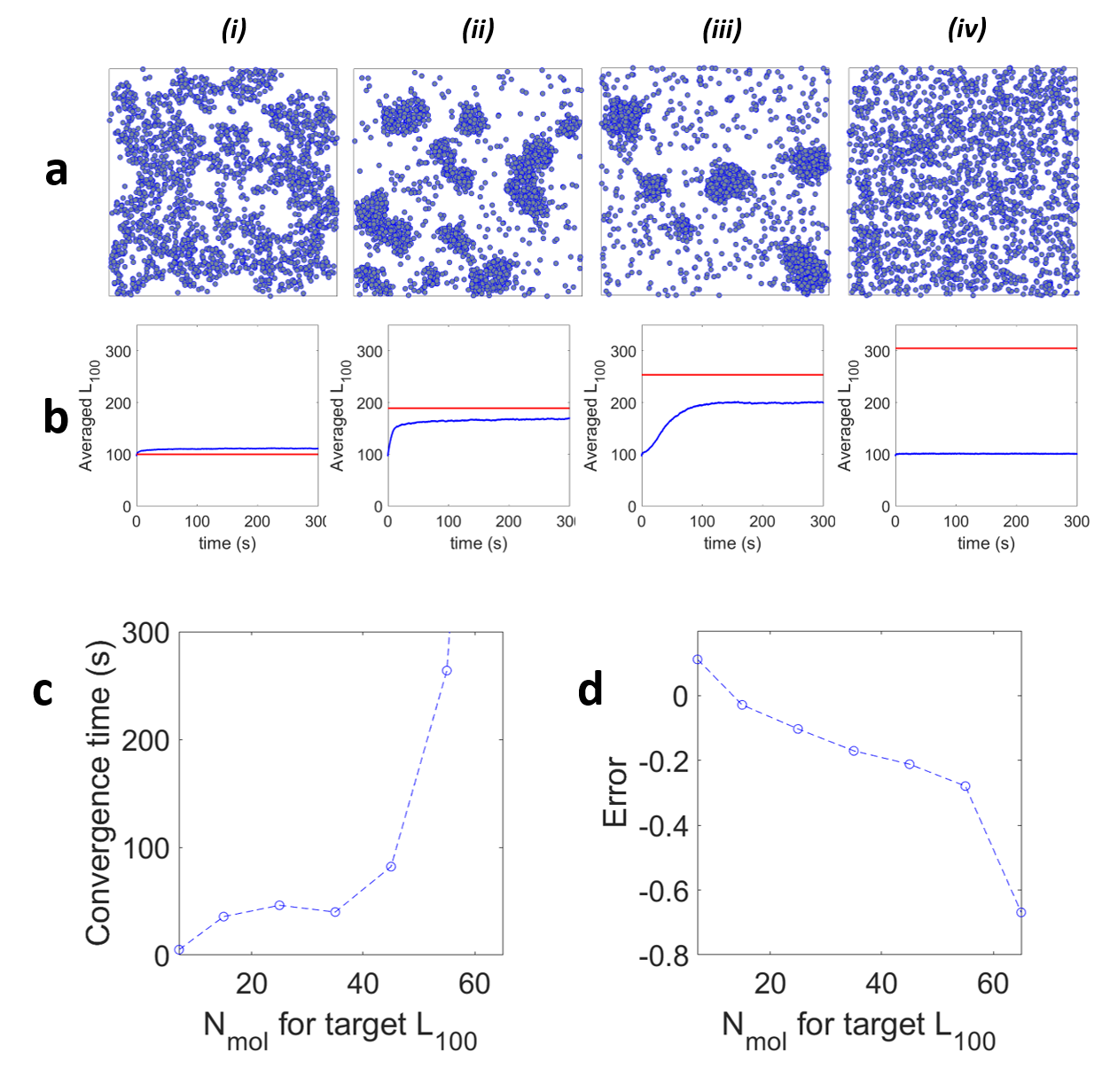

Supplement: S6 Fig — a. Examples of the molecular distributions obtained after 5 min simulations with the target values set at (i) 7, (ii) 25, (iii) 45, (iv) 55, (v) 65, encircled molecules. b. Corresponding <L^100> values extracted from all molecules over time (depicted blue) and their associated fixed targets (depicted red). c. Convergence time and d. Error as a function of the number of molecules for each L100 target (depicted in blue are the values obtained for each target). (TIF) [file pone.0226825.s006.tif]

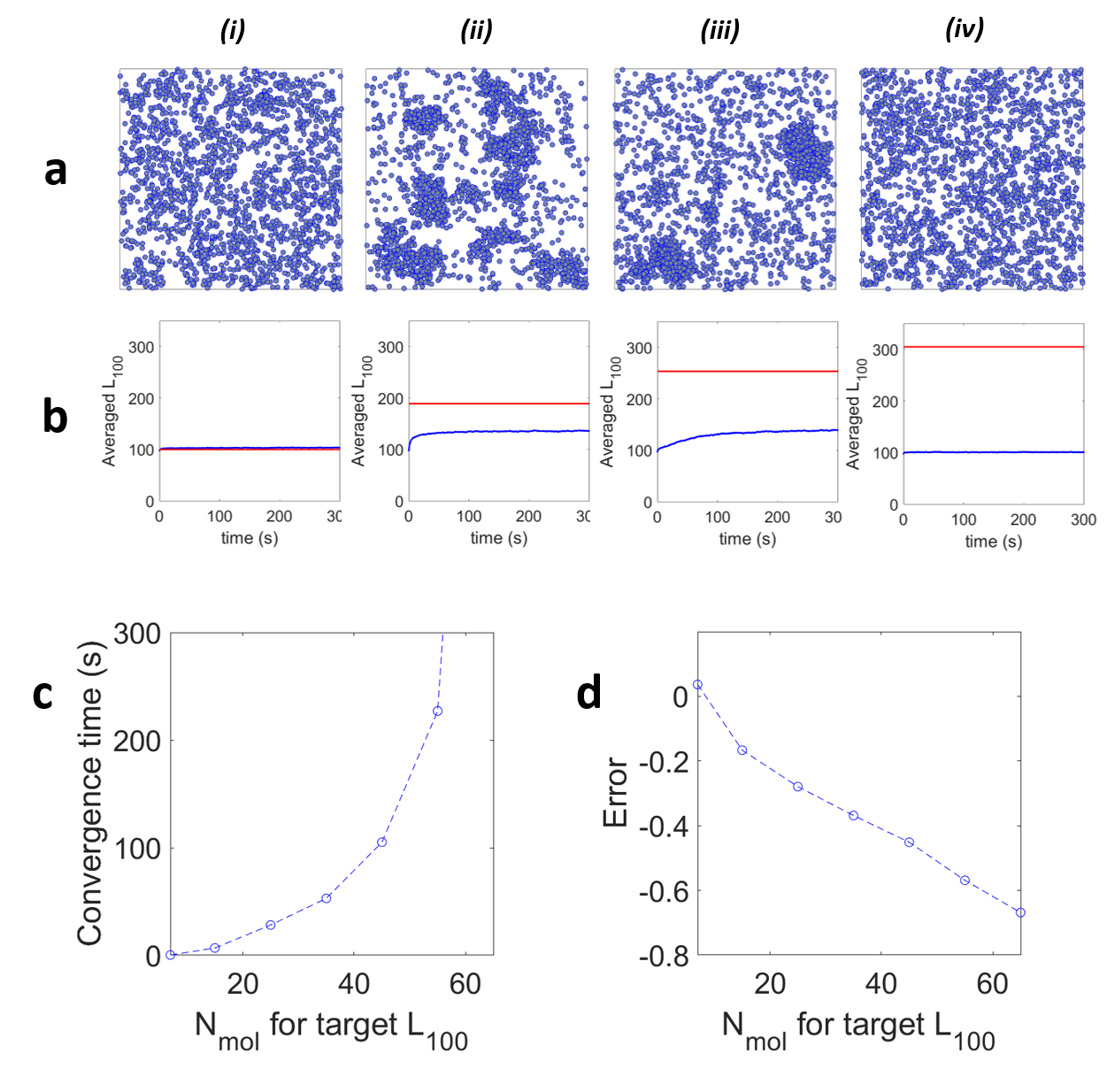

Supplement: S7 Fig — a. Examples of the molecular distributions obtained after 5 min simulations with the target values set at (i) 7, (ii) 25, (iii) 45, (iv) 55, (v) 65 encircled molecules. b. Corresponding <L^100> values extracted from all molecules over time (depicted blue) and their associated fixed targets (depicted red). c. Convergence time and d. Error as a function of the number of molecules for each L100 target (depicted in blue are the values obtained for each target). (TIF) [file pone.0226825.s007.tif]

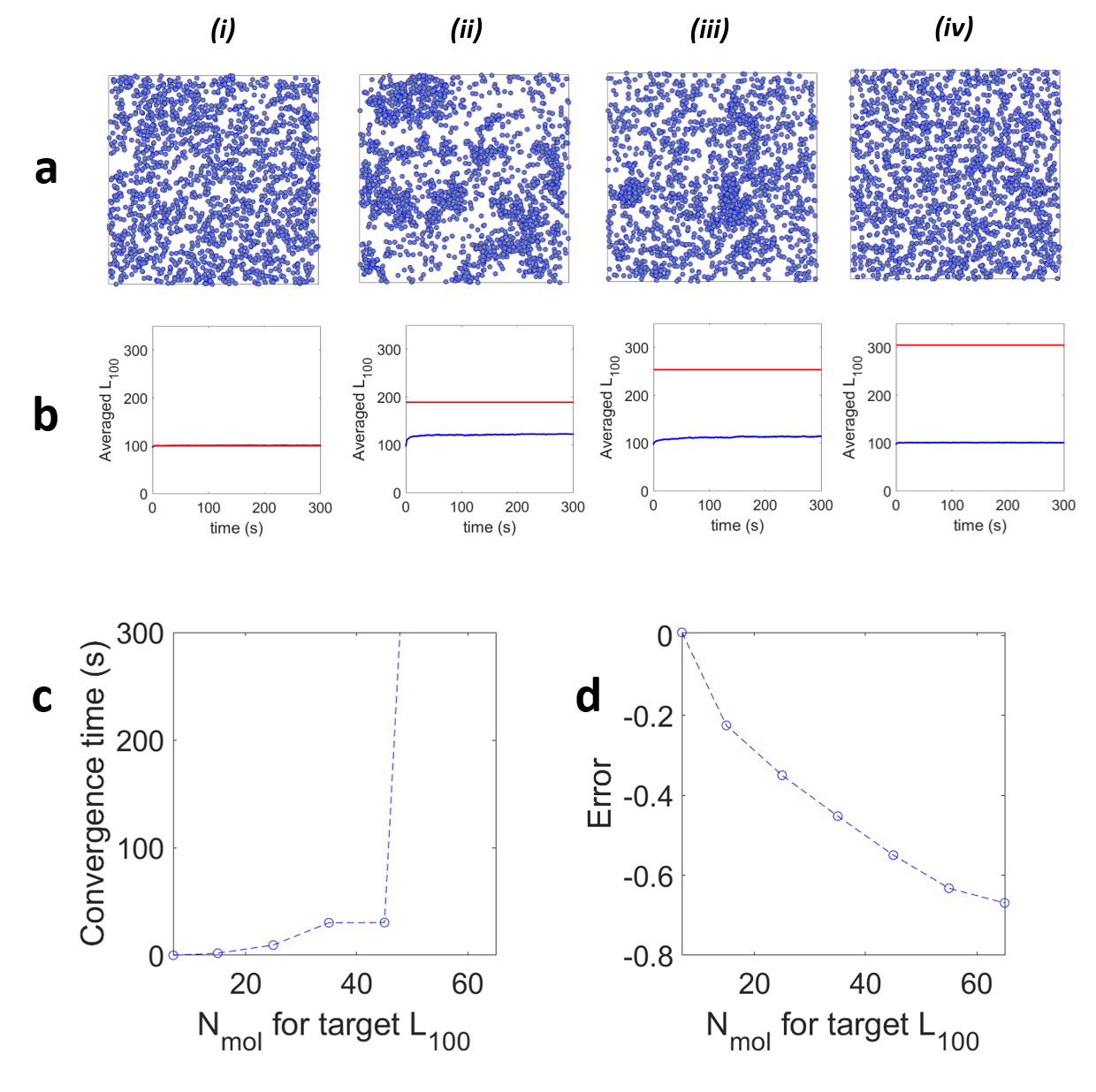

Supplement: S8 Fig — a. Examples of the molecular distributions obtained after 5 min simulations with the target values set at (i) 7, (ii) 25, (iii) 45, (iv) 55, (v) 65 encircled molecules. b. Corresponding <L^100> values extracted from all molecules over time (depicted blue) and their associated fixed targets (depicted red). c. Convergence time and d. Error as a function of the number of molecules for each L100 target (depicted in blue are the values obtained for each target). (TIF) [file pone.0226825.s008.tif]

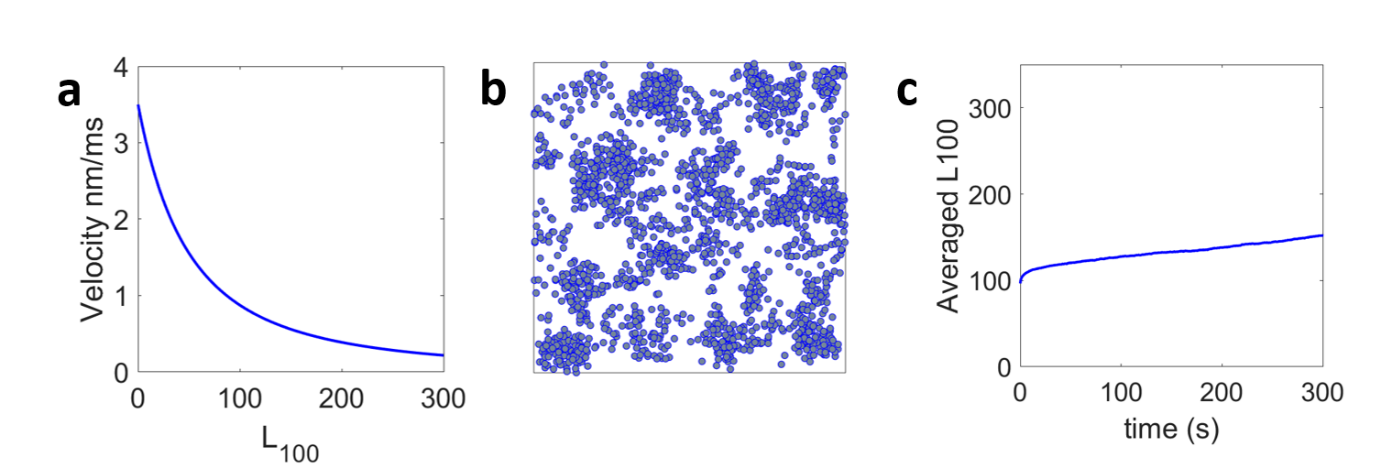

Supplement: S9 Fig — a. decay used for the displacement calculation of each molecule at each frame. b. Example of the molecular distributions obtained after 5 min. c. Corresponding <L^100> values extracted from all molecules over time (depicted blue). (TIF) [file pone.0226825.s009.tif]
